# Carbon fibres as potential bone implants with controlled doxorubicin release

**DOI:** 10.1038/s41598-022-06044-7

**Published:** 2022-02-16

**Authors:** Dorota Chudoba, Katarzyna Łudzik, Monika Jażdżewska

**Affiliations:** 1 Faculty of Physics, Adam Mickiewicz University, Poznan, 61-614 Poland; 2 Frank Laboratory of Neutron Physics, Joint Institute for Nuclear Research, Dubna, 141980 Russia; 3 Department of Physical Chemistry, University of Lodz, 90-236 Lodz, Poland

**Keywords:** Drug delivery, Nanoscience and technology, Nanostructures

## Abstract

This work presents the structural characterisation of carbon fibres obtained from the carbonization of flax tow at 400°C (CFs400°C) and 1000°C (CFs1000°C) and the thermodynamic and kinetic studies of adsorption of Doxorubicin (Dox) on the fibres. The characteristic of carbon fibres and their drug adsorption and removal mechanism were investigated and compared with that of natural flax tow. All fibres were fully characterized by scanning electron microscopy (SEM), Fourier transforms infrared spectroscopy (FT-IR), thermogravimetric analysis (TGA), specific surface area analysis and Boehm titration. The results demonstrated the highest adsorption properties of CFs400°C at 323 K (q_max_ = 275 mg g^−1^). The kinetic data followed the pseudo-second-order kinetic model more closely, whereas the Dubinin–Radushkevich model suitably described isotherms for all fibres. Calculated parameters revealed that the adsorption process of Dox ions is spontaneous and mainly followed by physisorption and a pore-filling mechanism. The removal efficiency for carbon fibres is low due to the effect of pore-blocking and hydrophobic hydration. However, presented fibres can be treated with a base for further chemical surface modification, increasing the adsorption capacity and controlling the release tendency.

## Introduction

For decades, carbon materials have been regarded as the most promising and useful products because of their important properties such as thermal stability, mechanical resistance, environment-friendliness, chemical inertness, bio and hemocompatibility^1–9^. The development of nanotechnology, fabrication and modification of carbon materials caused an enormous increase of scientific and industrial attention due to the new and broader spectrum of their potential applications that can improve the quality of life. A large share of works is those devoted to the use of carbon nanomaterials to biomedicine as nanocontainers^9–16^. The features of carbon nanomaterials such as large surface volume, porosity and functional surface meant that many types of drugs and antibodies could be chemically conjugated to their surface. Thus, carbon nanomaterial systems can deliver drugs and improve the stability of the drugs and prevent their uncontrolled uptake and removal. An additional advantage of carbon nanomaterials is that covalently modified carbon materials do not accumulate in organs but are prone to be excreted through urine^17^.

One promising candidate from a huge and various groups of carbon-based materials is produced from pyrolysis of organic precursor-like polyacrylonitrile or pitch in an inert atmosphere—carbon fibres, alternatively abbreviated as CFs^18,19^. These materials with a micro graphite crystal structure and low density, similar to bond density (1.6–2.2 g/cm^3^) exhibit unusual mechanical properties such as high specific strength or flexibility^19,20^. The superior properties in conjunction with electrical conductivity, high radiolucency, biocompatibility, inert nature and shape, that mimics the crystalline hydroxyapatite structures of natural bond, make the carbon fibres potential constituents of medical devices for structural fixation of skeleton fragments^3,20–26^. In addition, carbon fibres exhibit specific bioactivities in relation to the location in the living body. R. Petersen or K. Elias emphasize the ability of CFs to osseointegrate with live bone and reliability to stimulate tissue to grow and heal by removing respiratory stress products^20,24^. R. Price confirms dimension dependent cell adhesion on carbon filaments, which is crucial for dental or orthopaedic materials^26,27^. Although the surface of carbon filaments does not affect cell adsorption, it plays a crucial role in determining their end-use performance as a reinforcement for composite materials and effects on immunological cells response and adsorption ability^28^. Thus, controlled modification or functionalization of the surface of CFs can enhance properties and use those materials as a scaffold for tissues with simultaneously releasing drugs supporting regeneration or treatment.

It has been documented that the nature of precursor and selection of carbonization parameters affect structure and mechanical properties of carbon fibres^29,30^. The type and degree of activation determine its physical and chemical properties.

In this study, we characterized the structure of three types of fibres: flax tow—pristine fibres pFs, fibres after carbonization at 400°C (CFs400°C), fibres after carbonization at 1000°C (CFs1000°C) and investigated from the kinetic and thermodynamic point of view process of adsorption and release of doxorubicin hydrochloride (Dox). A better understanding of the interaction mechanism of Dox with carbon fibres as well as kinetic and thermodynamic of adsorption and release of the drug will provide opportunity to realize the most effective strategy for their surface modification and in consequence exhibits the opportunity to use the material as a bone implants with controlled Dox  release.

## Experimental

### Materials

Carbon fibres (CFs) were prepared from a natural precursor—flax tow (pFs) at Warsaw University. The pristine materials are in the form of carbonaceous fibres with a diameter below 100 µm. The chemical composition of flax is similar to other bio-resources. The flax is a mixture of polymerized units of glucose (lignin, pectin and hemicelluloses). The surface modification included thermal annealing at two different temperatures: 400°C (CFs400°C) and 1000°C (CFs1000°C). The thermal treatment causes carbonization and changes the surface chemistry. The process was carried out in a tube furnace equipped with a gas matrix (inert atmosphere Ar to eliminate possible oxidation processes) with a heating rate of 5 °C min^−1^. Afterwards, the furnace was cooled down naturally. Doxorubicin hydrochloride (purity ≥ 98%, Sigma Aldrich) -Dox, was used without further purification. All solutions were prepared in Milli-Q (The Millipore ultrapure water Co., Ltd., Millipore, Burlington, MA, USA) ultrapure water.

### Samples characterisation

Surface chemical features were studied by FT-IR spectroscopy (Thermo Fisher Nicolet iS5) and EDX spectrometry (Zeiss Merlin equipped with EDX spectrometer). FT-IR measurements were carried out in a transmission mode with a spectral resolution of 4 cm^−1^. A little amount of the studied sample (ca. 1 mg) was mixed with 300 mg KBr and pressed in a hydraulic press. The content of surface acidic groups was determined from Boehm titration by using Metrohm automatic titrator Titrando 808.

Scanning electron microscopy was used to determine morphological features (Zeiss Merlin equipped with EDX spectrometer). Before measurements, the studied samples were covered by a thin conductive layer.

The porous structure of the samples was evaluated by physical adsorption of N_2_ at 77 K in a Micromeritics ASAP 2020 apparatus. By using the Brunauer-Emmitt-Teller equation the specific surface area was determined. Pore volumes were estimated at a relative pressure of 0.94 p/p^0^, assuming full surface saturation with nitrogen. Pore size distributions were evaluated from desorption branches of nitrogen isotherms using the Barret–Joyner–Halenda (BJH) model.

Thermogravimetric analyzer TA Instruments TA Q50 was used for the characterization of pure CFs and CFs with adsorbed doxorubicin (the heating rate of 5 deg min^−1^ and nitrogen atmosphere).

Dox adsorption efficiency and drug release processes were investigated spectroscopically by means of UV–Vis: Shimadzu 2401 spectrophotometer as described previously^31^.

### Equilibrium adsorption studies

The isotherms of Dox on investigated carriers were determined as follows. A known mass of the material was added to the solution containing various initial concentrations of doxorubicin in PBS buffer (2–700 mg L^−1^). The as-obtained mixtures were shaken for 24 h. Next, the decrease of Dox concentration was determined spectrophotometrically using a standard curve method^32^. The data obtained in equilibrium studies were used to calculate the equilibrium Dox adsorptive quantity by using the following mass balance:

1 $$q_{e} = \frac{{\left( {C_{0} - C_{e} } \right)V}}{m}$$

where: *q*_*e*_ is the amount of Dox adsorbed at equilibrium (mg g^−1^), *V* is the volume of solution treated (L), *C*_*o*_ is the initial concentration of Dox (mg L^−1^), *C*_*e*_ is the equilibrium Dox concentration (mg L^−1^) and *m* is the mass of the adsorbent (g).

The adsorption studies were carried out at three constant temperatures: 298.15 K 310.15 K and 323.15 K. All experiments were conducted in triplicate and the mean values have been reported. The equilibrium data were fitted with the Langmuir, Freundlich, Dubinin–Radushkevich (D-R) equilibrium adsorption isotherm models^33–38^.

### Adsorption kinetics studies

The adsorption kinetic curve of doxorubicin onto pristine and surface modified materials were determined similarly to the determination of adsorption isotherms. The adsorption kinetics was determined for the initial concentration of doxorubicin of 100 mg mL^−1^. The mixtures were shaken for the specified time interval (5–15 min). The maximum contact time was 120 min. Finally, the decrease of Dox concentration was determined spectrophotometrically using a standard curve method^31^. The amount of drug adsorbed at time t, q_t_ (mg g^−1^), was calculated by the Eq. (2):

2 $$q_{t} = \frac{{\left( {C_{0} - C_{t} } \right)V}}{m}$$

where *C*_*t*_ (mg L^−1^) is the concentration of Dox at any time *t*.

### Studies on release of doxorubicin

The acetic buffer was chosen as the liquid medium for the studies on the doxorubicin release. The buffer was prepared by dissolving 0.1 mol (8.2 g) of sodium acetate in 1 L of 0.1 M acetic acid solution. The resulting pH was measured and it was equivalent to 4.95–5.05. The release of Dox from all pristine and surface modified materials was as follows: ca. 10 mg of the materials with adsorbed Dox was suspended in the specified volume of the medium and shaken for 24 h. Next, the solid material was separated by filtration or centrifugation. The concentration of Dox was determined spectrophotometrically using a standard curve method.

The kinetics of release of doxorubicin in acetic buffers was studied by preparation of several mixtures. In each one ca. 10 mg of the material with adsorbed Dox was placed in the acetic buffer. The volume of this medium was 50 mL. The mixtures were shaken for specified time intervals. Again, the concentration of released doxorubicin was determined spectrophotometrically. The kinetic results of Dox release process were presented as a function of % Dox released in time *t* Eq. (3):

3 $$release\; \% \;Dox = \frac{{C_{t} }}{{C_{0} }} 100\%$$

where *C*_*t*_—concentration of drug released in *t* time, *C*_*0*_—maximum drug concentration obtained during the release process.

## Results

### Fibres characterisation

**Figure 1:**
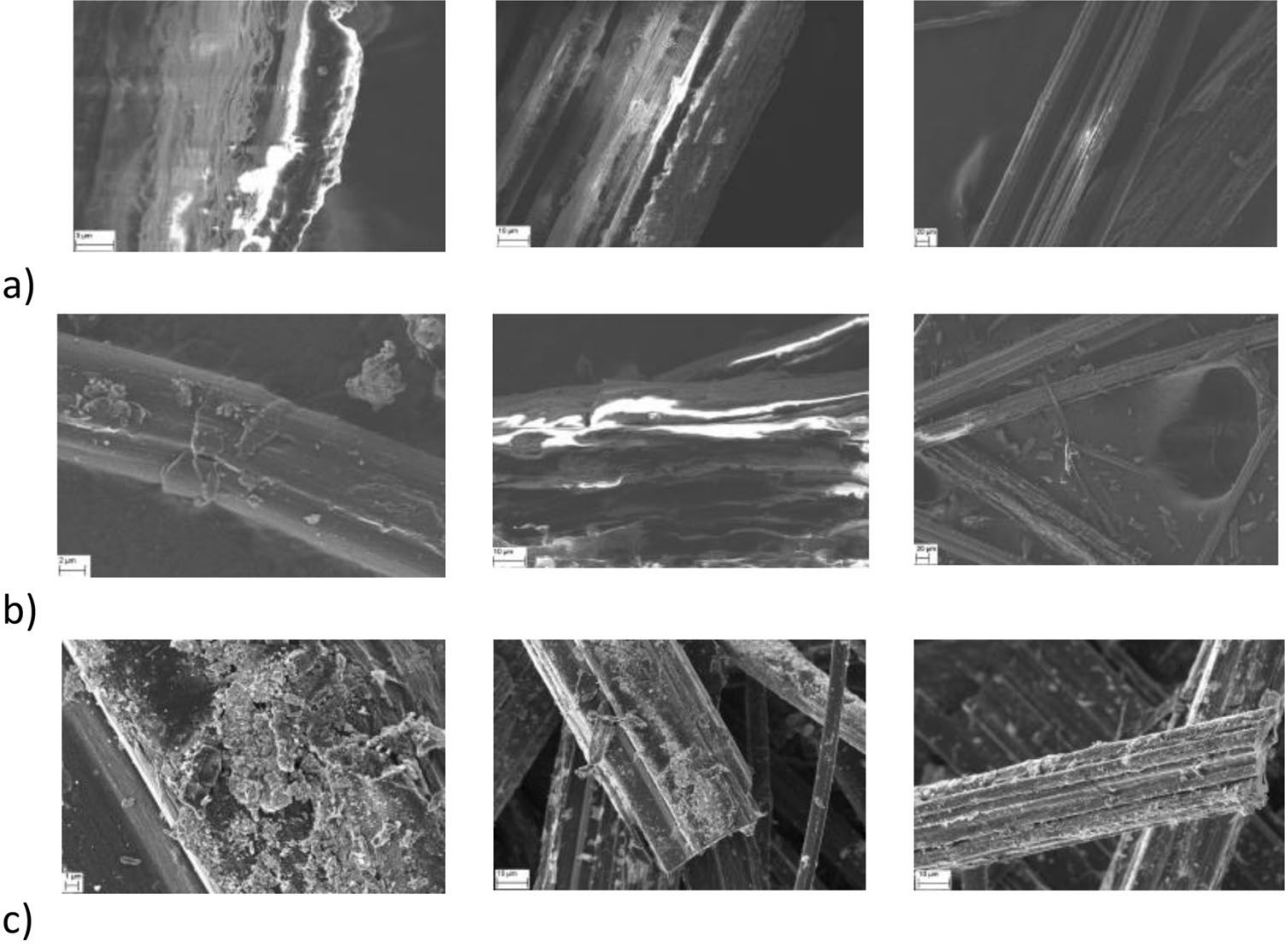
SEM images of: pure flax tow (in scale from left: 3µm, 10µm, 20µm)  (**a**); carbonaceous flax fibres obtained after having been heated at 400 °C (in scale from left: 2µm, 10µm, 20µm)   (**b**) and of carbonaceous flax fibres obtained after having been heated at 1000°C (in scale from left: 1µm, 10µm, 10µm) (**c**).

The morphological analysis of used filaments was carried to elucidate the adsorption and release mechanisms of doxorubicin on/from the fibres. The representative microscopy images of three types of investigated fibres: flax tow, fibres after carbonization at 400°C and at 1000°C were presented in Fig. 1a–c respectively. It is well visible that all the samples exhibit “fibrous” morphology. The rough surface is noticeable in case of CFs1000°C. The diameter of obtained fibres varies between 8 to 80 µm. The shape of the fibres after carbonisation is oblate Fig. 1c (on the right).

The shape of the TGA curve for flax tow is typical and reflects the volatilization of water and adsorbed substances (100–250°C) and then char formation. TGA curves of investigated fibres determined under non-oxidizing atmosphere are presented in the supplementary materials—Figure S1. In the case, CFs400°C mass, the loose mass observed on the TGA curve can be attributed to the decomposition and thermal degradation of functional groups. In addition, the wide range of temperatures of loose mass suggests a different type of labile oxygen-containing groups on the CFs400°C surface. Similarly, the smooth shape of the TGA curve in case CFs1000°C confirms the marginal amount of functional groups on the fibre surface and the high thermal stability of the sample. In order to investigate the types of acidic groups, Boehm titration was applied. The result is presented in the supplementary materials—Table S1.

**Figure 2:**
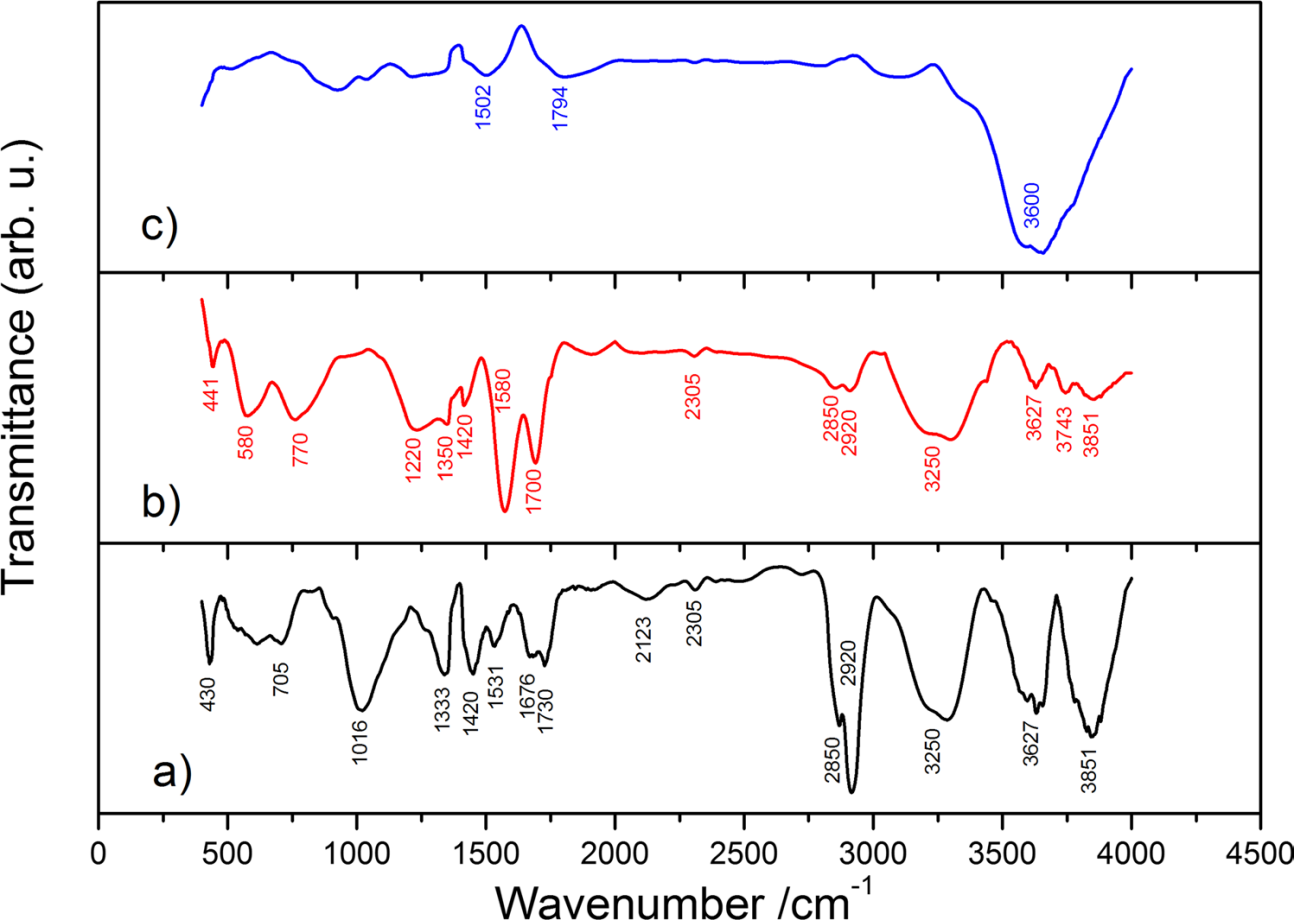
Fourier transform infrared spectroscopy (FT-IR) spectra of: (**a**) pFs, (**b**) CFs400°C (**c**) CFs1000°C.

Types of functional groups were also determined based on Fourier transform infrared spectroscopy (FT-IR) Fig. 2.

As is well seen in Fig. 2c, for CFs1000°C sample the broad peak at 3600 cm^−1^ reflects the stretching vibration of –OH groups. In case CFs400°C—Fig. 2b, peaks from 3600–3800 cm^−1^ can be attributed to stretching vibration of not associated OH groups. The peak at 3250 cm^−1^ is assigned to stretching vibration of able to create hydrogen bonds -OH groups. The peaks at 2850 and 2920 cm^−1^ indicate stretching vibration of C-H groups. Regarding the peaks at 1700, 1580, 1400, 1420, 1350, 1220 cm^−1^ it can be said that indicates the existence of asymmetric and symmetric stretching vibration of C = O groups, stretching vibration of aromatic C = C groups and stretching C-O groups. Obtained spectra of CF400°C confirms the existence of phenolic and lactonic groups that was concluded from Bohem titration. Small peaks from range 580–770 cm^−1^ are due to bending vibrations of –OH groups^39,40^.

Characterisation of the surface area of fibres was carried out by using nitrogen adsorption/desorption. The main textural parameters of investigated fibres were collected in the supplementary materials Table S2. It shows that pFs and CFs400°C practically do not contain micropores. What is more, the volume of narrow mesopores (2–10 nm) does not exceed 0.009 cm^3^ g^−1^ , which is also marginal. As a result, the specific surface area for both samples is low. This is an expected finding because pF and CFs400°C have a rather non-porous surface presented on SEM images. Carbonization at 1000°C causes an increase of porosity (mainly micropores 0.031 cm^3^ g^−1^) and develops the specific surface area Fig. 1c.

### Doxorubicin adsorption and release

**Figure 3:**
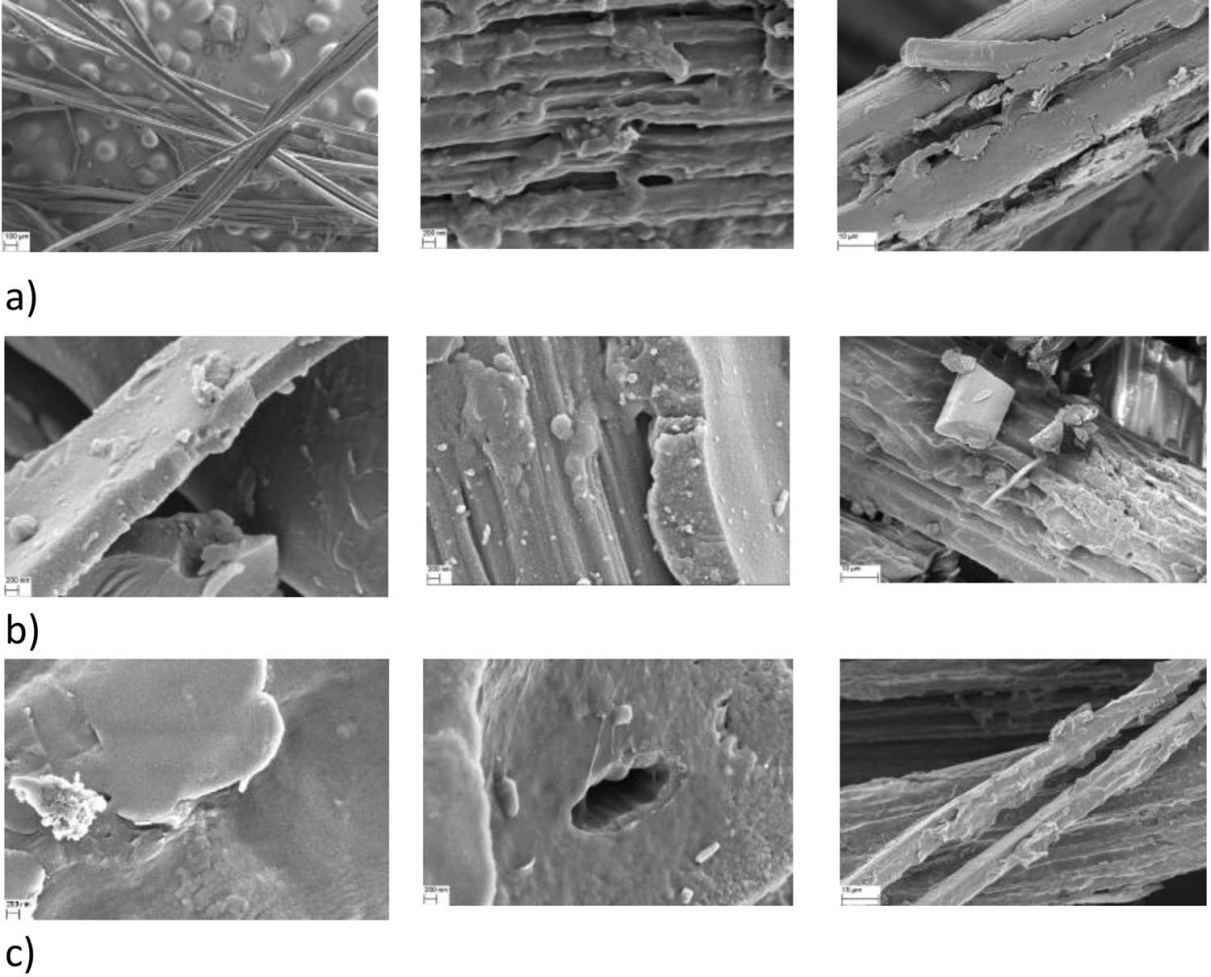
SEM images of: pure flax fibres with adsorbed molecules of Dox (in scale from left: 100µm, 200nm, 10µm) (**a**); carbonaceous flax fibres obtaining after having been heated at 400 °C with adsorbed molecules of Dox (in scale from left: 200nm, 200nm, 10µm) (**b**); carbonaceous flax fibres obtaining after having been heated at 1000°C with adsorbed molecules of Dox (in scale from left: 200nm, 200nm, 10µm)  (**c**).

The characterisation of a career with Dox was carried out by using SEM, TGA, FT-IR techniques and nitrogen adsorption tests. SEM results presented in Fig. 3 confirm that doxorubicin molecules have been grafted on the surface of carbon fibres. It is worth noticing that the adsorption of the drug is more efficient in case of pFs and CFs400°C than CFs1000°C.

From the TGA measurements (presented in the supplementary materials Figure S2) it can be stated that fibres with Dox yield more weight loss than pure fibres, which is attributed to the degradation of organic components of the vulnerable under such a high thermal condition drug.

The textural characteristic after adsorption and pore distribution before and after the drug adsorption were analysed to understand the mechanism of doxorubicin adsorption and its relationship with surface structure. The results were presented in the supplementary materials Table S3 and Figure S3.

Adsorption of Dox exhibits pore-size dependent tendency. The change in the pore size suggests that molecules of Dox do not occupy micropores lower than 4  nm. It is worth noticing that materials with very low porosity (S_BET_ < 10 m^2^ g^−1^) can exhibit artefacts on the curves.

### Adsorption isotherm and thermodynamic studies

**Figure 4:**
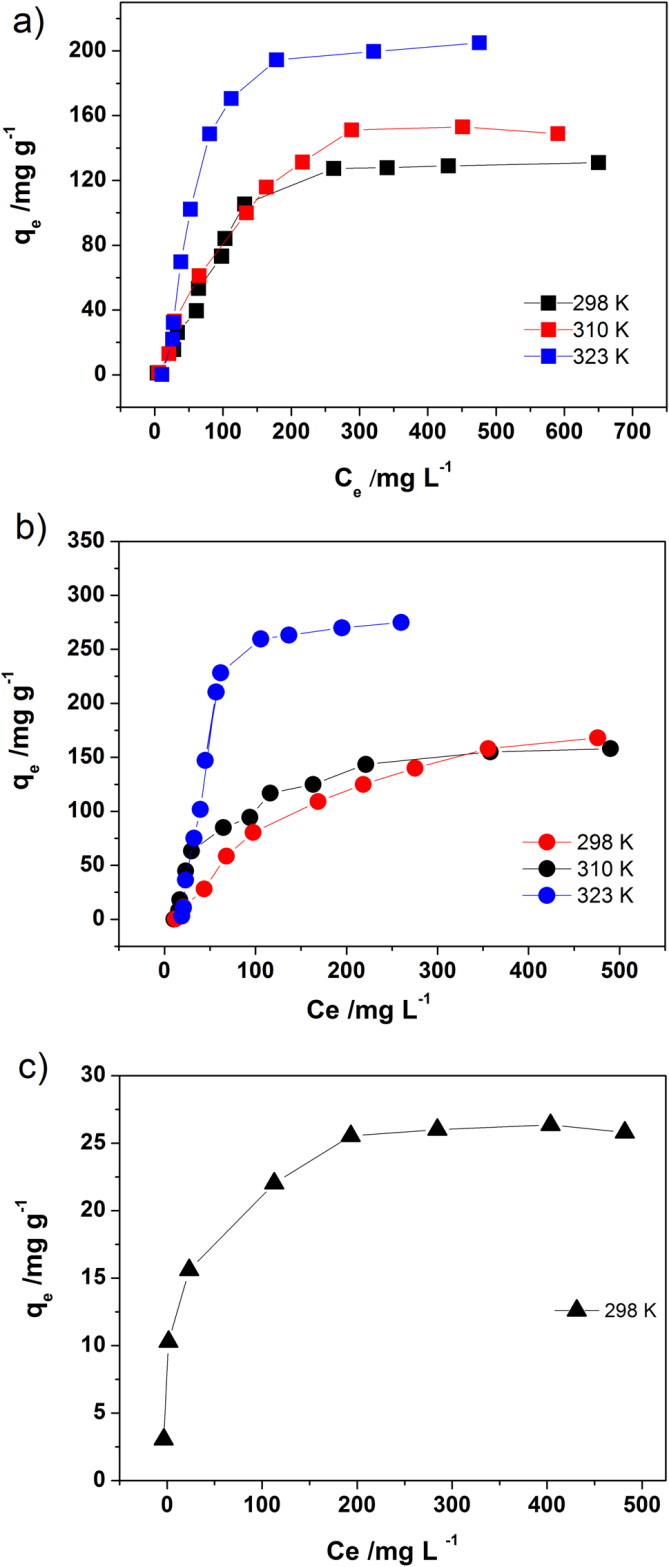
Adsorption isotherms of Dox on (**a**) pFs; (**b**) CFs400°C; (**c**) CFs1000°C.

In order to explore the process of Dox adsorption on fibres in more depth three equilibrium adsorption isotherm models: Freundlich, Langmuir and Dubinin–Radushkevich were applied to the adsorption equilibrium data presented in Fig. 4. Carbon fibres obtained at 1000°C have much more unsatisfactory adsorption performance in comparison to the pristine fibres and CFs400°C sample. Therefore, further studies of adsorption were not carried out on that sample.

The earliest known relationship describing adsorption on heterogonous surfaces with active sites with different energy is Freundlich isotherm^37,41^:

4 $$q_{e} = K_{F} C_{e}^{1/n}$$

where: *q*_*e*_ is the amount of Dox adsorbed per unit weight of adsorbent at equilibrium (mg g^−1^), *C*_*e*_ the equilibrium concentration of Dox in the bulk solution (mg L^−1^), *K*_*F*_ is adsorption capacity mg^1−1/n^ L^1/n^ g^−1^ and *1/n* is heterogeneity factor.

Langmuir isotherm assumes that adsorption takes place at a finite number of identical and energetically equivalent sites and the adsorbed layer is one molecule in thickness of monolayer adsorption^36^. The Langmuir equation can be described by Eq. (5):

5 $$q_{e} = \frac{{q_{m} K_{L} C_{e} }}{{1 + K_{L} C_{e} }}$$

where: *qe* is the amount of drug adsorbed per unit weight of adsorbent at equilibrium (mg g^−1^), *C*_*e*_—the equilibrium concentration of Dox in the bulk solution (mg L^−1^), *q*_*m*_ the maximum adsorption capacity (mg g^−1^), and *K*_*L*_ is the constant related to the free energy of adsorption (L mg^−1^).

Adsorption mechanism on heterogeneous surfaces that follows a pore-filling mechanism can be described by temperature-dependent semi-empirical equation proposed by Dubinin Radushkevich^38^:

6 $$q_{e} = q_{m} exp\left[ { - \beta \left( {RTln\left( {1 + \frac{1}{{C_{e} }}} \right)} \right)^{2} } \right]$$

where: *q*_*e*_ is the maximal amount of drug adsorbed per unit weight of adsorbent at equilibrium (mg g^−1^), *C*_*e*_ the equilibrium concentration of Dox in the bulk solution (mg L^−1^), *q*_*m*_ (mg g^−1^) is a constant related to adsorption capacity, *R*—gas constant, *T* is absolute temperature, *β* (g^2^ kJ^−2^ ) is a constant related to the mean free energy of adsorption – *E* (kJ g^−1^):

7 $$E = \frac{1}{{\sqrt {2\beta } }}$$

The magnitude of *E* is the main criterion for determining the type of adsorption mechanism. The value of the free energy < 8 kJ mol^−1^ suggests physical adsorption^42,43^, the value of *E* in the range 8- 16 kJ/mol, indicates the adsorption process follows ion exchange^44^. Values of *E* higher than 20 kJ/mol indicates chemisorption^45,46^. The constant parameters of the two-parameter isotherm models were calculated by nonlinear regression analysis using Origin 9 and presented in Table 1.

**Table 1:** Freundlich, Langmuir and Dubinin-Radushkevich isotherm parameters obtained by nonlinear fitting for the three types of fibres: pFs, CFs400°C and CFs1000°C for: 298, 310, 323 K.

Fiber	The value of parameters for isotherm model		
	Freundlich	Langmuir	Dubinin–Radushkevich
pFs	T = 298 K		
	K_F_ = 9.126	q_m_ = 172.8	q_m_ = 131.4
	n = 2.280	K_L_ = 0.0076	r^2^ = 0.979
	r^2^ = 0.847	r^2^ = 0.944	E = 5.9
	T = 310 K		
	K_F_ = 9.826	q_m_ = 198.7	q_m_ = 148.0
	n = 2.208	K_L_ = 0.0077	r^2^ = 0.984
	r^2^ = 0.881	r^2^ = 0.973	E = 5.8
	T = 323 K		
	K_F_ = 15.220	q_m_ = 269.5	q_m_ = 200.8
	n = 2.231	K_L_ = 0.0104	r^2^ = 0.994
	r^2^ = 0.768	r^2^ = 0.901	E = 7.9
CFs400°C	T = 298 K		
	K_F_ = 4.178	q_m_ = 200.2	q_m_ = 133.7
	n = 1.626	K_L_ = 0.0102	r^2^ = 0.973
	r^2^ = 0.949	r^2^ = 0.980	E = 14.0
	T = 310 K		
	K_F_ = 11.039	q_m_ = 266.3	q_m_ = 172.4
	n = 2.210	K_L_ = 0.0039	r^2^ = 0.969
	r^2^ = 0.870	r^2^ = 0.959	E = 5.36
	T = 323 K		
	K_F_ = 17.214	q_m_ = 417.6	q_m_ = 290.1
	n = 1.892	K_L_ = 0.0108	r^2^ = 0.982
	r^2^ = 0.665	r^2^ = 0.775	E = 10.5
CFs1000°C	T = 298 K		
	K_F_ = 9.878	q_m_ = 27.2	q_m_ = 26.8
	n = 6.037	K_L_ = 0.0550	r^2^ = 0.950
	r^2^ = 0.962	r^2^ = 0.958	χ^2^ = 3.885
	χ^2^ = 2.974	χ^2^ = 0.748	E = 2.3

q_m_/mg g^−1^; E/kJ mol^−1^.

Table 1 shows that the Dubinin-Radushkevich isotherm model can generate a satisfactory fit to the experimental data for pFs, CFs400°C and CFs1000°C within all investigated temperature ranges. Moreover, obtained from D-R model values of q_m_ are comparable with the experimental q_max_ (Table S4 supplementary materials) and correspond to the adsorption isotherm plateau (Fig. 4).

That confirms the modelling of D-R for the adsorption system is acceptable. The calculated value of free sorption energy for the CFs1000°C and pFs (E < 8 kJ mol^−1^) presented in Table 1 indicates that physisorption played a significant role in the adsorption process at measured temperature range. For CFs400°C value of E > 8 kJ mol^−1^ suggests that adsorption can follow not only physisorption and a pore-filling mechanism but also through ion exchange. It can be explained by the presence of functional groups present on the surface that can react with drug molecules. The best adsorption properties of Dox exhibit CFs400°C. Obtained results stay in good agreement with pore distribution that indicate the CFs400 °C surface as the most potential for adsorption (mesopores). The rough surface of CFs1000°C exhibits a weak adsorption capacity. It can be explained by the presence of micropores the size of which does not correspond to the size needs of Dox as well as the marginal number of functional groups. The influence of temperature on Dox sorption on fibres is the most significant for temperature 323 K. Similar results were noticed in the case of paracetamol and ibuprofen on activated carbon^47–49^. The explanation of the increase of efficiency of the adsorption process for higher temperatures can be explained by the solvation of drug molecules in an aqueous solution. The hydration shell is a barrier in the adsorption process and has to be removed. At higher temperatures, the thermic motions cause that the hydration sheaths are less stable, and the dehydration phenomenon occurs freely^50^.

Thermodynamic functions as a standard change in Gibbs free energy ∆G°, the standard change in enthalpy ∆H°, and the standard change in entropy ∆S° form the basis of understanding the nature of the adsorption process. The thermodynamic equilibrium constant for the adsorption process K_0_ allowed to determined ∆G° by using Eq. (8):

8 $$\Delta G^{o} = - RTlnK_{0}$$

where *K*_*0*_ is equal to *q*_*e*_*/C*_*e*_ and was determined from the intercept of *ln(q*_*e*_*/C*_*e*_*)* vs. *q*_*e*_ at temperature *T* (K), *R* is the universal gas constant.

The average standard enthalpy and entropy change for pFs and CFs400 °C were estimated from the slope and intercept of the linear form of the van 't Hoff equation:

9 $$lnK_{0} = \frac{{\Delta S^{0} }}{R} - \frac{{\Delta H^{0} }}{RT}$$

Obtained parameters were presented in the supplementary materials in Table S5. Due to the poor adsorption performance of CFs1000°C in comparison to the CFs400°C, further studies on CFs1000°C were not carried out.

The adsorption process of Dox on the fibres is spontaneous; however, the amount of adsorbed drugs stays varied Table S5. It is worth noticing that the feasibility of adsorption as well as the experimental maximum adsorption capacity Q_max_ is the highest for CFs400°C. Positive values of ∆S° suggest an irregular increase of randomness at the adsorbent/solution interface during the adsorption of the drug molecules on the carrier^51^. A similar tendency was observed in the case dyes adsorption on cation-exchange resin^52^ and Dox adsorption onto graphene oxide^53^.

The value of the average standard change in enthalpy can be interpreted in terms of the type of adsorption. G. Bayramoglu and co-authors^52^ suggest that the value of ∆H° from the range between − 20 to − 40 kJ/mol reflects the physical adsorption whereas more negative values of ∆H° (range between − 400 and − 80 kJ/mol) reveals the prevalent contribution of chemisorption. Therefore, it can be said that obtained positive values of ∆H° confirm that the physical nature of the uptake dominates. The higher value of ∆H° obtained for CFs400°C reveals stronger interaction between Dox and CFs400°C than in case of pFs and as a result, leads to enhanced adsorption.

### Kinetic studies of Dox adsorption and release

**Figure 5:**
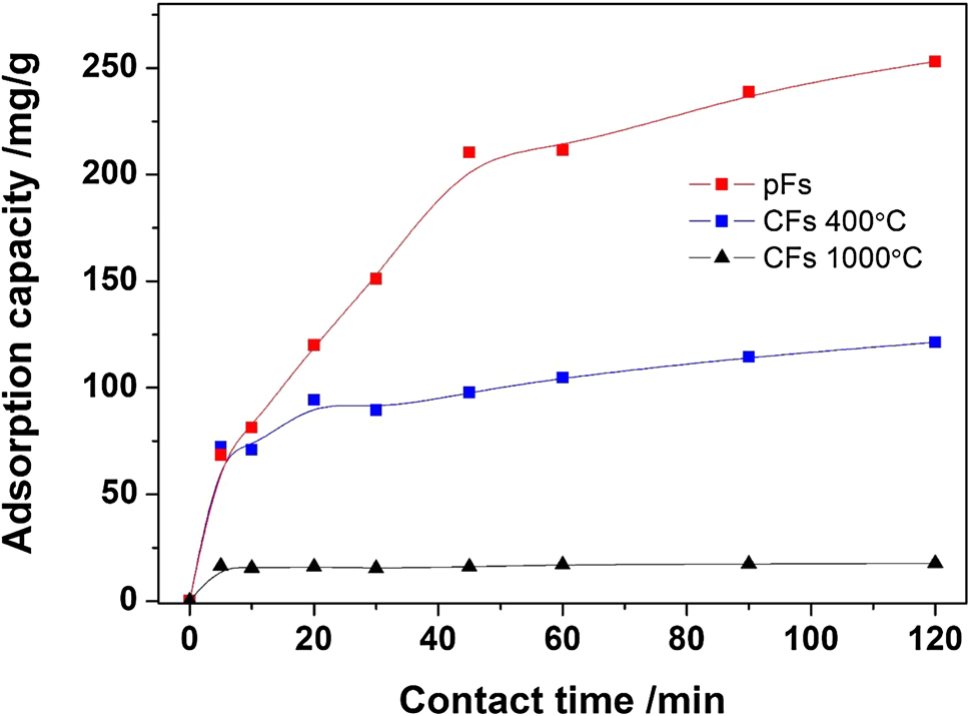
Influence of contact time on Dox adsorption capacity pF, CFs400 °C and CFs1000°C.

On the basis of the experimentally obtained adsorption kinetic curve in Fig. 5, it can be suggested that the adsorption process of Dox on CFs1000°C is very rapid and after 5 min reaches the almost maximal value of adsorption capacity equal ~ 17 mg g^−1^. For CFs400°C and pFs the equilibrium is reached after 100 min; however, the adsorption process is more efficient compared to CF1000°C, which indicates the equilibrium adsorption capacity equal ~ 120 mg g^−1^ and 250 mg g^−1^ respectively Fig. 5.

 Two kinetic models, which are pseudo-first-order (Lagergren, 1898) Eq. (10) and pseudo-second-order (Ho and McKay, 1999) Eq. (11) were applied to the experimental data^53–55^.

10 $$log\,log \left( {q_{e} - q_{t} } \right) = logq_{e} - \frac{{k_{1} }}{2.303}t$$

where: *k*_*1*_ is the Lagergren rate constant of adsorption /min^−1^, *q*_*e*_ and *q*_*t*_ denote the amounts of adsorption at equilibrium and at time *t* /mg g^−1^, respectively.

11 $$\frac{t}{{q_{t} }} = \frac{1}{{k_{2} q_{e}^{2} }} + \frac{t}{{q_{e} }}$$

where: *k*_*2*_ is the pseudo second-order rate constant of adsorption /g mg^−1^ min^−1^, *q*_*e*_ and *q*_*t*_ denote the amounts of adsorption at equilibrium and at time *t* /mg g^−1^, respectively.

The best fit and comparable values of experimental and calculated values of *q*_*e*_ were obtained for the pseudo second order kinetic model. Parameters of pseudo first and pseudo second kinetic models were presented in supplementary materials in Table S6.

Values of k_2_ confirms that the adsorption rate changes in sequence: CFs1000°C  > CFs400°C  > pFs.

**Figure 6:**
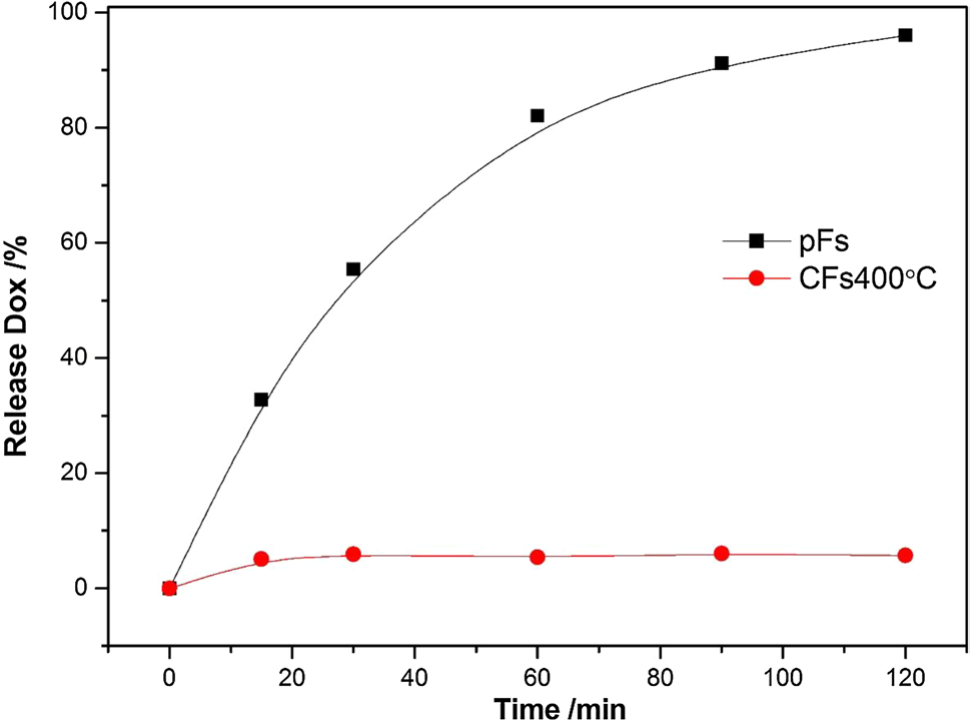
The release of Dox from pFs and CFs400°C at 298 K.

The kinetic results of the Dox release process are presented in Fig. 6. For CFs1000°C the percentage of release did not exceed 2% therefore the results were not presented.

Release of Dox accumulated on CF400°C does not exceed 6%. Low release of doxorubicin (~ 5%) was noticed for ion exchange microspheres at 298 K. However, temperatures of 316 K had up to 3 times higher release rates than 298 K^56^. Drug release kinetics was used to determine the Dox release mechanism and best-fit drug release model. In this study, zero order, first order, second order, Higuchi, Weibull, and Korsmeyer–Peppas/Ritger-Peppas kinetic models were applied^57^.

The zero-order kinetics model is expressed by the equation:

12 $$C_{t} = C_{0} + K_{0} t$$

where: *C*_*t*_ is the concentration of the drug released over time *t*, *C*_*0*_ is the concentration of the drug in solution before release, and *K*_*0*_ is the zero-order release rate constant.

The first-order kinetics model:

13 $$ln\left( {C_{d} - C_{t} } \right) = ln\left( {C_{d} } \right) - K_{1} t$$

where *C*_*t*_ is the concentration of the drug released over time *t*, *C*_*d*_ is the concentration of the drug before dissolution and *K*_*1*_ in the equation is the first-order release rate constant.

The second-order kinetics model represented by the equation:

14 $$\frac{1}{{\left( {C_{d} - C_{t} } \right)}} = \frac{1}{{C_{d} }} - K_{2} t$$

where *C*_*t*_ is the concentration of the drug released over time *t*, *C*_*d*_ is the initial concentration of the drug and *K*_*2*_ is the second-order rate constant.

Higuchi model considering a low concentration of the active agent in the matrix, where the solubility and release happen through the porosity of the matrix is expressed as:

15 $$C_{t} = K_{H} t^{\frac{1}{2}}$$

where *C*_*t*_ is the concentration of the drug released at time *t* and *K*_*H*_ is the Higuchi rate constant.

Weibull model represented by the equation:

16 $$C_{m} = C_{s} \left[ {1 - e^{{ - \frac{{\left( {t - T} \right)^{b} }}{a}}} } \right]$$

where *C*_*m*_ is the concentration of Dox dissolved as a function of time *t*, *C*_*s*_ is the total concentration of Dox being released, *T* means the latency time of the release process, *a* is the scale parameter which defines the timescale of the process and *b* characterizes the curves as either exponential (*b* = 1) S-shaped (*b* > 1) or parabolic (*b* < 1).

Korsmeyer–Peppas (Power Law) and Ritger-Peppas semi-empirical model:

17 $$C = \frac{{C_{t} }}{{C_{\infty } }} = Kt^{n}$$

where *C* is the released concentration of the drug, *C*_*t*_ is the concentration of drug released over time *t*, *C*_*∞*_ is the concentration of drug at the equilibrium state, *K* is kinetic constant (having units of t^n^) incorporating structural and geometric characteristics of the delivery system, *n* is the diffusion exponent, which related to the drug release mechanism, in the function of time *t*.

The results of the application of different mathematical models to in-vitro drug release from pFs and CFs400°C were represented in Table 2.

**Table 2:** Parameters obtained from the kinetic models release of Dox from pFs and CFs400°C.

Parameters			
CFs400°C		pFs	
**The zero-order**			
K_0_/mg^−1^ min^−1^	0.0097	K_0_/g mg^−1^ min^−1^	0.6571
r^2^	0.8506	r^2^	0.8717
**The first-order**			
K_1_/min^−1^	7.09 × 10^–5^	K_1_/min^−1^	0.0269
r^2^	0.9453	r^2^	0.9985
**The second-order**			
K_2_/ml g^−1^ min^−1^	6.718 × 10^–07^	K_2_/ml g^−1^ min^−1^	0.00169
r^2^	0.905	r^2^	0.8599
**Higuchi**			
K_H_/mg ml^−1^ min^−1/2^	0.1303	K_H_/mg ml^−1^ min^−1/2^	10.203
r^2^	0.9559	r^2^	0.9452
**Weibull**			
b	0.0617	b	1.013
r^2^	0.9273	r^2^	0.999
**Power law**			
K	0.04436	K	0.1154
n	0.0607	n	0.4504
r^2^	0.905	r^2^	0.958

Dox accumulated on CFs400°C and pFs display the first-order release. Many therapeutic systems display the first-order release for example betamethasone adsorbed on implants^58^ or ibuprofen cumulated on carboxymethyl cellulose/mesoporous magnetic graphene oxide^59^. The release mechanism of Dox from the polylactide spheres also followed the first-order^60^.

The high degree of the correlation coefficient for the Higuchi kinetic model suggests that the prime mechanism of Dox release from the non-swellable matrix is diffusion controlled. Diffusion exponent n ≤ 0.5 obtained from Power-law model also implies Fickian diffusion release Table 2.

The release of Dox from the functionalized mesoporous silica nanoparticles was also described by Thashini Moodley and Moganavelli Singh as undergoing rapid Fickian diffusion with no friction effects^61^.

In our opinion, the drug release process consists of two steps: the first rapid release called “burst of drug molecules” adsorbed at the surface (physisorbed by electrostatic and π–π dispersion interactions), and the second: drug release through pores. The release of covalently attached Dox depends on pH or temperature^60,61^. The pH influence can be attributed to hydrogen bonding strength that decreases for the acidic environment and as result prompt the drug release. Thus, we expect that the release of covalently attached Dox in acidic pH (~ 5) occurs relatively fast. The weak drug release tendency for CFs400°C can be caused not only by the pore-blocking effect but mainly by hydrophobic hydration of career that hinders the water movement and consequently diffusion and drug release.

## Conclusion

The findings of this study indicated that the best adsorption properties of Dox exhibit CFs400°C, although the specific surface area is very low (~ 13 m^2^ g^−1^). It shows that molecules of Dox can locate only in pores which dimension higher than 4 nm. The increasing temperature induces the dehydration of the drug molecules that facilitate the adsorption that demonstrates visibly higher adsorption efficiency at 323 K for all investigated systems.

Adsorption of Dox is spontaneous for all fibres and positive values of ∆H° indicate that the physical nature of the uptake dominates. The main contribution of physisorption confirms also values of free sorption energy calculated based on the Dubinin-Radushkevich isotherm model. In case CFs400°C results obtained show that adsorption follows not only physiosorption and a pore-filling mechanism but also through ion exchange. It can be explained by the presence of functional groups (phenolic mainly) determined by Boehm Titration and FT-IR spectra.

The kinetic models show that the prime mechanism of Dox release is diffusion controlled. The release of drugs from carbon fibres at 298 K is hindered. The reason for this fact can be the effect of pore-blocking as well as hydrophobic hydration of the fibres that obstruct the diffusion process.

In summary, obtained carbon fibres exhibit average adsorption and weak release of Dox. However, there are many possible ways of modifying their surface due to increase the drug adsorption and control the drug release. Thus, fibres can be considered as a material with significant potential for medical applications. What is more, a long time release is worth investigating.

## Supplementary Information

Supplementary Figures.

Supplementary Tables.
