# Supplementary material for: Carbon fibres as potential bone implants with controlled doxorubicin release
Source: Sci Rep. 2022 Feb 16;12:2607. doi: 10.1038/s41598-022-06044-7 (PMC8850544; doi:10.1038/s41598-022-06044-7)
Supplement: Supplementary file 1 — Supplementary Figures. [file 41598_2022_6044_MOESM1_ESM.docx]

**Figure S1**. TGA curves of investigated fibres determined under non-oxidizing atmosphere.

**Figure S2**. TGA curves of investigated fibres with Dox in combination with TGA curves of materials without drug.

a)

b)

c)

**Figure S3**. Pore size distribution for: pFs before and after adsorption of DOX (a); CFs400°C before and after adsorption of DOX (b) and CFs1000°C before and after adsorption of DOX (c).
