# Supplementary material for: Carbon fibres as potential bone implants with controlled doxorubicin release
Source: Sci Rep. 2022 Feb 16;12:2607. doi: 10.1038/s41598-022-06044-7 (PMC8850544; doi:10.1038/s41598-022-06044-7)
Supplement: Supplementary file 2 — Supplementary Tables. [file 41598_2022_6044_MOESM2_ESM.docx]

**Table S1**. Content of surface acidic groups determined from Boehm titration.

| Functional group | CFs 400°C | CFs 1000°C |
| --- | --- | --- |
|  | concentration on surface  /mmol g^-1^ | concentration on surface  /mmol g^-1^ |
| Carboxylic /mmol g^-1^ | 0.00 | 0.02 |
| Phenolic  /mmol g^-1^ | 1.24 | 0.02 |
| Lactonic  /mmol g^-1^ | 0.38 | 0.04 |
| Sulfonic  /mmol g^-1^ | 0.00 | 0.00 |

**Table S2**. Textural characteristic of studied fibres: S_BET_ – specific surface area, V_micro_ - micropore volume, V_meso_ - mesopore volume (pore size range between 2 and 10 nm), V_total_ - total pore volume.

|  | pFs | CFs 400°C | CFs100°C |
| --- | --- | --- | --- |
| S_BET_  /m^2^ g^-1^ | 49 | 13 | 86 |
| V_micro_  /cm^3^ g^-1^ | 0.0002 | 0.0001 | 0.0310 |
| V_mezo_  /cm^3^ g^-1^ | 0.0023 | 0.0087 | 0.0061 |
| V_total_  /cm^3^ g^-1^ | 0.0032 | 0.0097 | 0.0393 |

**Table S3**. Textural characteristic of materials after doxorubicin adsorption: SBET – specific surface area, Vmicro - micropore volume, Vmeso - mesopore volume (pore size range between 2 and 10 nm), Vtotal - total pore volume.

|  | pFs | CFs 400°C | CFs100°C |
| --- | --- | --- | --- |
| S_BET_  /m^2^ g^-1^ | 9.2 | 49 | 40 |
| V_micro_  /cm^3^ g^-1^ | 0.0009 | 0.0069 | 0.0030 |
| V_mezo_  /cm^3^ g^-1^ | 0.0019 | 0.0018 | 0.0109 |
| V_total_  /cm^3^ g^-1^ | 0.0029 | 0.1394 | 0.0882 |

**Table S4**. Experimental maximum adsorption capacity qmax for pFs, CFs400°C, CF1000°C determined at: 298 K, 310K and 323 K.

| T /K | pFs | CFs 400°C | CFs1000°C |
| --- | --- | --- | --- |
|  | q_max_  / mg g^-1^ | q_max_  / mg g^-1^ | q_max_  / mg g^-1^ |
| 298 | 130 | 158 | 26 |
| 310 | 150 | 170 | - |
| 323 | 205 | 275 | - |

**Table S5**. Thermodynamic parameters for DOX adsorption on pFs, CFs400°C and CFs1000°C.

| pFs | CFs400°C | CFs1000°C |
| --- | --- | --- |
| **T=298K** | | |
| K_0_ = 1.0816  ∆G^°^=-0.19 | K_0_ = 1.4780  ∆G^°^=-1.01 | K_0_ =1.0900  ∆G^°^=-0.23 |
| **T=310K** | | |
| K_0_ =1.5968  ∆G^°^=-1.21 | K_0_ = 3.9709  ∆G^°^=-3.42 |  |
| **T=323K** | | |
| K_0_ =6.3547  ∆G^°^=-4.97 | K_0_ = 16.0916  ∆G^°^=--7.46 |  |
| ∆H^°^= 56.68  ∆S^°^= 189.5 | ∆H^°^= 76.43  ∆S^°^= 259.1 |  |

∆G^°^/ kJ mol^-1^; ∆H^°^/ kJ mol^-1^; ∆S^°^/ J mol^-1^ K^-1^

**Table S6.** Parameters of pseudo first and pseudo second kinetic models.

| pFs | | CFs400°C | CFs1000°C |
| --- | --- | --- | --- |
| Pseudo First Order | | | |
| q_e_ 274.3  k_1_ 0.006991  r^2^ 0.9599 | q_e_ 147.9  k_1_ 0.001476  r^2^ 0.9487 | | q_e_ 10.9  k_1_ 0.007946  r^2^ 0.7468 |
| Pseudo Second Order | | | |
| q_e_ 303.03  k_2_ 0.000131  r^2^ 0.9867 | q_e_ 126.1  k_2_ 0.000911  r^2^ 0.9922 | | q_e_ 17.86  k_2_ 0.02100  r^2^ 0.9983 |
